# Episodic Abdominal Pain Characteristics Are Not Associated with Clinically Relevant Improvement of Health Status After Cholecystectomy

**DOI:** 10.1007/s11605-016-3156-5

**Published:** 2016-05-17

**Authors:** Mark P. Lamberts, Wietske Kievit, Jos J. G. M. Gerritsen, Jan A. Roukema, Gert P. Westert, Joost P. H. Drenth, Cornelis J. H. M. van Laarhoven

**Affiliations:** Scientific Institute for Quality of Healthcare (IQ healthcare), Radboud University Medical Centre, PO Box 9101, 6500 HB Nijmegen, The Netherlands; Department of Gastroenterology and Hepatology, Radboud University Medical Centre, Nijmegen, The Netherlands; Department of Surgery, Radboud University Medical Centre, Nijmegen, The Netherlands; Department for Health Evidence, Radboud University Medical Centre, Nijmegen, The Netherlands; Department of Surgery, Medisch Spectrum Twente, Enschede, The Netherlands; Department of Surgery, St. Elisabeth Hospital, Tilburg, The Netherlands

**Keywords:** Pain, Health status, Cholecystectomy

## Abstract

**Background:**

Cholecystectomy is the therapy of first choice in patients with uncomplicated symptomatic cholecystolithiasis, but it remains unclear which patients truly benefit in terms of health status improvement. Patients generally present with episodic abdominal pain of varying frequency, duration, and intensity. We assessed whether characteristics of abdominal pain episodes are determinants of clinically relevant improvement of health status after cholecystectomy.

**Methods:**

In a post hoc analysis of a prospective multicenter cohort study, patients of ≥18 years of age with uncomplicated symptomatic cholecystolithiasis subjected to cholecystectomy were included. Preoperatively, patients received a structured interview and a questionnaire consisting of the visual analogue scale (VAS; range 0–100) and gastrointestinal quality of life index (GIQLI). At 12 weeks after cholecystectomy, the GIQLI was again administered. Logistic regression analyses were performed to determine significant associations.

**Results:**

Questionnaires were sent to 261 and returned by 166 (63.6 %) patients (128 females, mean age at surgery 49.5 ± 13.8). A total of 131 (78.9 %) patients reported a clinically relevant improvement of health status. The median (interquartile range) frequency, duration, and intensity of abdominal pain episodes were 0.38 (0.18–0.75) a week, 4.00 (2.00–8.00) hours, and 92 (77–99), respectively. None of the characteristics was associated with a clinically relevant improvement of health status at 12 weeks after cholecystectomy.

**Conclusions:**

Characteristics of abdominal pain episodes cannot be used to inform patients with symptomatic cholecystolithiasis who are skeptic about the timing of cholecystectomy for optimal benefit. Timing of cholecystectomy should therefore be based on other characteristics and preferences.

## Introduction

Cholecystolithiasis represents a clinical spectrum that ranges from asymptomatic gallstone disease to uncomplicated symptomatic gallstone disease to acute cholecystitis. Likewise, therapeutic options may go from conservative treatment to cholecystectomy. Patients with asymptomatic cholecystolithiasis benefit least from cholecystectomy in terms of improvement of health status and should receive conservative care, whereas those with acute cholecystitis benefit most and should receive surgery.^1^–^4^ The optimal timing that results in the highest benefit for patients with uncomplicated symptomatic cholecystolithiasis remains less clear.

Uncomplicated symptomatic cholecystolithiasis is frequently characterized by abdominal pain episodes of widely varying frequency. These episodes may last minutes or several hours, and their intensity is variable.^5^ Previous studies have shown that patients with a higher frequency of episodic abdominal pain were less likely to obtain pain relief after cholecystectomy.^6^^–^10 Conversely, those with a typical episode duration between 30 min and 24 h were more likely to report absence of pain after surgery, whereas patients with a higher pain intensity were not more likely to report absence of pain.^10^ Abdominal symptom characteristics may also indicate which patients are most likely to display clinically relevant improvement of patient-reported overall health status after cholecystectomy: a more comprehensive outcome measure that not only includes symptom evaluation, but also emotional, physical, and social functioning.^11^

We aimed to assess whether frequency, maximum duration, or intensity of abdominal pain episodes were associated with improvement of health status in order to define which patients with uncomplicated symptomatic cholecystolithiasis may benefit most from cholecystectomy. We also assessed the associations of these episode characteristics with different subscales of health status at 12 weeks after cholecystectomy.

## Methods

### Study Sites and Patient Selection

We performed a post hoc analysis using the database established during a previous multicenter cohort study conducted in the Netherlands. Details of study design were reported previously.^12^ In short, all individuals aged 18 years and over with symptomatic cholelithiasis, who visited the surgical outpatient clinic at a tertiary referral center (Radboud University Medical Centre, Nijmegen) or one of the two non-academic teaching hospitals (St. Elisabeth Hospital, Tilburg and the Medisch Spectrum Twente Hospital, Enschede) between June 2012 and June 2014, and were scheduled for elective cholecystectomy were eligible for participation in the study. Cholelithiasis was defined as abdominal pain associated with gallstones, confirmed with ultrasound imaging. Medical histories were obtained by a single physician (MPL) through a structured interview.

Patients were asked to recall the duration of symptoms, the number of episodes, and longest episode duration. Patients with a history of symptoms for more than 1 year or who reported to have experienced more than five episodes were excluded, because most of these patients could not recall the frequency. Consequently, these data were only sporadically reported in the database. In addition, we excluded those with schizophrenia or other mental disorders that may impair recall. Other exclusion criteria were as follows: a history of complicated symptomatic cholelithiasis (acute cholecystitis, cholangitis, biliary pancreatitis, choledocholithiasis requiring endoscopic retrograde cholangiopancreatography (ERCP)),^13^,^14^ ASA fitness grades III and IV, insufficient knowledge of the Dutch language, non-Dutch residency, blindness, pregnancy, cirrhosis, or cancer treatment. Eligible patients were asked to complete a questionnaire before cholecystectomy and 12 weeks after cholecystectomy. Patients who failed to return or complete the questionnaire before and after surgery were excluded.

The questionnaire consisted of the gastrointestinal quality of life index (GIQLI). The GIQLI has been developed in Germany and has been translated and validated in Dutch.^15^,^16^ For an example of the questions and response categories of this questionnaire, we refer to a previous study.^15^ The GIQLI addresses upper and lower gastrointestinal symptoms (19 questions), emotional (5 questions), physical (7 questions), social well-being (4 questions), and effect of medical treatment (1 question) in the previous 2 weeks. Each question contains five response categories. Questions can be scored using a response scale ranging from 0 (worst appraisal) to 4 points (best appraisal) for each question, giving an overall score of 0–144 points. The higher the score, the better overall health status is. A clinically relevant improvement after surgery was defined as an increase of 5 points or more in the overall score or in any of the subscales.^17^ We also included a visual analogue score (VAS) providing a range of scores from 0 to 100 to quantify the maximum severity of pain preoperatively.

The study was approved by the medical ethics committee and reported in accordance with the recommendations in the Strengthening the Reporting of Observational studies in Epidemiology (STROBE) guidelines for reporting observational studies.^18^

### Outcomes and Variables of Interest

The primary outcome was defined as a clinically relevant improvement of overall health status. Secondary outcomes included a clinically relevant improvement of upper and lower gastrointestinal symptoms, or on the emotional, physical, and social subscales, respectively. Based on previous publications, the independent variables included sex,^10^,^19^ age at operation,^10^,^19^ center,^10^ baseline GIQLI score,^19^ ASA fitness grade, frequency, maximum duration, and intensity of abdominal pain episodes.

### Statistical Analysis

We examined whether baseline clinical and abdominal pain characteristics differed between responders and non-responders to the questionnaire, using *χ*^2^ tests or Fisher’s exact tests for categorical data, Student’s *t* tests for continuous data, and Mann-Whitney *U* tests for ordinal data. We determined which variables were associated in univariable analysis with a clinically relevant improvement of health status after surgery using logistic regression analyses. Significant variables in univariable analysis (*P* < 0.10) were introduced into a backward multivariable regression model to determine whether there were independent predictors of clinically relevant improvement of overall health status or any of the subscales after surgery. Age at operation, sex, center, and baseline GIQLI score were the variables that were retained in the model as co-variables. Results were reported as adjusted odds ratios (ORs) with corresponding 95 % confidence intervals. *P* < 0.05 was considered statistically significant. All missing values were considered to be completely at random and excluded from analyses. Statistical analyses were performed using SPSS statistical software version 20.0 (IBM, Armonk, NY, USA).

## Results

**Fig. 1:**
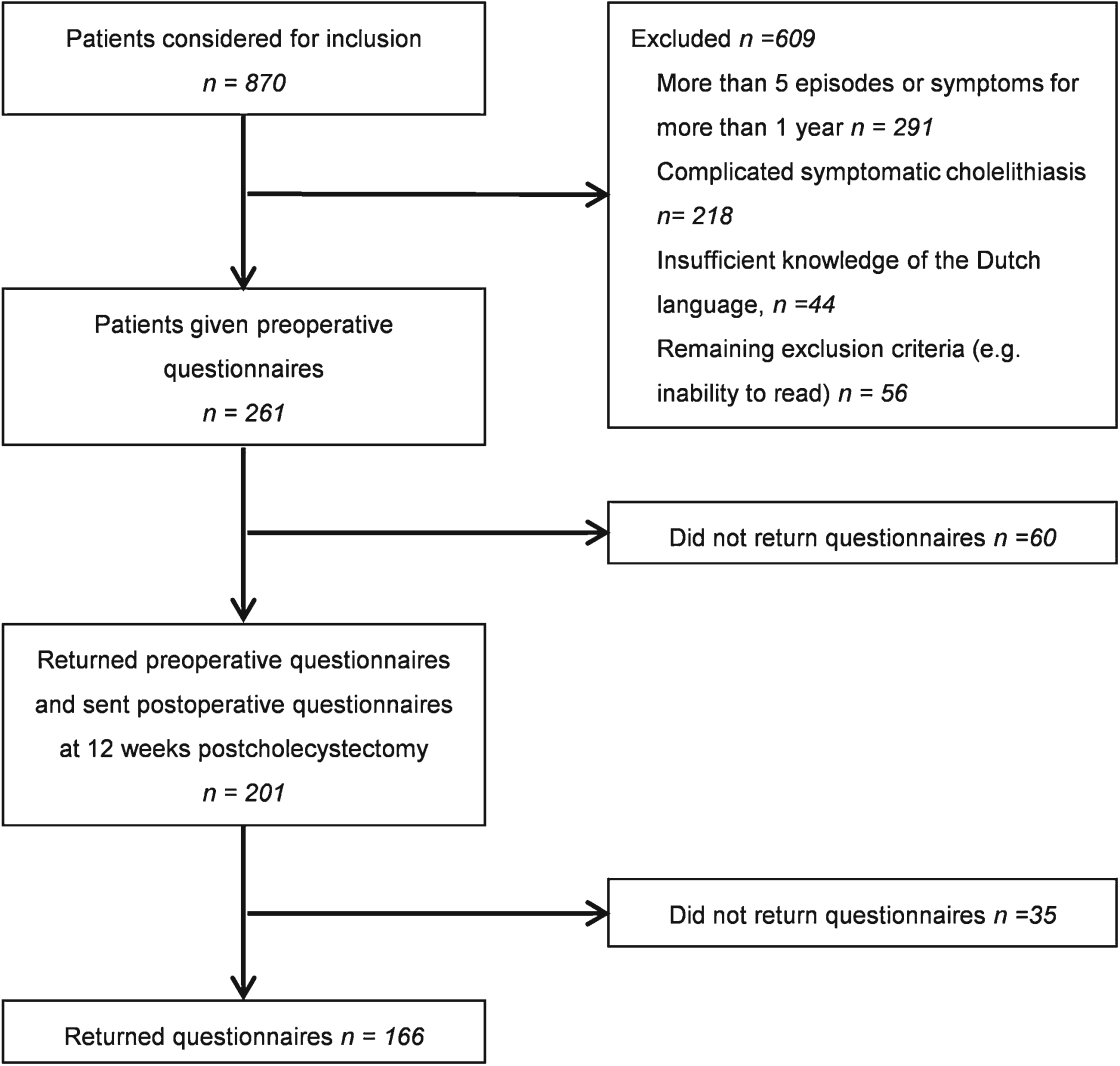
Flow chart showing inclusion of patients in the study

The database consisted of 870 potentially eligible patients. A total of 261 patients were included. Preoperative and postoperative questionnaires were returned and completed by 166 (63.6 %) patients (Fig. 1). Baseline characteristics of the responding patients are shown in Table 1. One hundred and twenty-eight of the responding patients were females. Mean age at surgery was 49.5 ± 13.8 years. The median (interquartile range) frequency, duration, and intensity of abdominal pain episodes were 0.38 (0.18–0.75) a week, 4.00 (2.00–8.00) hours, and 92 (77–99), respectively. Baseline and abdominal pain characteristics did not differ between responders and non-responders.

**Table 1:** Characteristics of the responding and non-responders to the questionnaires

Characteristic	Responders, *n* = 166	Non-responders, *n* = 95	*P* value
Age (years)	49.5 ± 13.8	46.3 ± 16.3	0.09
Sex			0.81
Male	38 (22.9)	23 (24.2)	
Female	128 (77.1)	72 (75.8)	
ASA fitness grade			0.46
I	83 (50.0)	52 (54.7)	
II	83 (50.0)	43 (45.3)	
Center			
Radboud UMC	36 (21.7)	19 (20.0)	NA
MST	69 (41.6)	47 (49.5)	NA
St Elisabeth Hospital	61 (36.7)	29 (32.2)	NA
Frequency of abdominal pain episodes a week	0.4 (0.2–0.8)	0.3 (0.2–0.7)	0.65
Duration of longest abdominal pain episode in hours	4.0 (2.0–8.0)	4.0 (2.0–6.3)	0.48
Maximum intensity of pain episode ranging 0–100	92 (77–99)		NA
Baseline GIQLI score	103.5 ± 22.1		NA
GIQLI score 12 weeks after cholecystectomy	124.4 ± 13.7		NA

Data are expressed as mean (standard deviation) or *n* (%) or median (interquartile range)

*ASA* American Society of Anesthesiologists, *GIQLI* gastrointestinal quality of life index, *UMC* University Medical Center, *NA* not applicable, *MST* Medisch Spectrum Twente hospital

One hundred and thirty-one (78.9 %) patients reported an overall clinically relevant improvement after surgery. Univariable analysis showed maximum intensity of abdominal pain episodes to be associated with clinically relevant improvement of overall health status (OR 1.02, 95 % CI 1.00–1.04; *P* = 0.069) (Table 2). Maximum intensity of abdominal pain episodes did not remain associated in multivariable analysis (OR 1.03, 95 % CI 1.00–1.05; *P* = 0.066).

**Table 2:** Univariable and multivariable association of pain episode characteristics with patient-reported minimal clinically important improvement of health status

	Clinically relevant improvement		Univariable analysis		Multivariable analysis	
	<5 points on GIQLI, *n* = 35	≥5 points on GIQLI, *n* = 131	Odds ratio (95 % CI)	*P* value	Odds ratio (95 % CI)	*P* value
Age (years)	50.6 ± 13.6	49.2 ± 13.9	0.99 (0.97–1.02)	0.586	1.0 (0.96–1.04)	0.885
Sex				0.179		0.650
Female	24 (68.6)	104 (79.4)	1.77 (0.77–4.05)		0.76 (0.24–2.46)	
Male	11 (31.4)	27 (20.6)	1.00 (reference)			
Hospital type				0.850		0.812
Tertiary referral center	8 (22.9)	28 (21.4)	0.92 (0.38–2.24)		0.86 (0.24–3.06)	
Non-academic	27 (77.1)	103 (78.6)	1.00 (reference)			
Baseline GIQLI score	126.6 ± 11.8	97.3 ± 20.0	0.88 (0.84–0.92)	<0.001	0.88 (0.84–0.92)	<0.001
ASA fitness grade				0.568		
II	16 (45.7)	67 (51.1)	1.24 (0.59–2.63)			
I	19 (54.3)	64 (48.8)	1.00 (reference)			
Frequency of pain episodes a week	0.4 (0.2–0.5)	0.4 (0.2–1.0)	1.66 (0.65–4.26)	0.287		
Maximum duration of longest pain episode in hours	4.0 (2.5–9.0)	4.0 (2.0–8.0)	1.01 (0.96–1.07)	0.648		
Maximum intensity of pain episode ranging 0–100	88.5 (72.0–94.3)	93.5 (79.0–99.8)	1.02 (1.00–1.04)	0.069	1.03 (1.00–1.05)	0.066

Data are expressed as mean (standard deviation) or *n* (%) or median (interquartile range)

*95* % *CI*, 95 % confidence interval, *ASA* American Society of Anesthesiologists, *GIQLI* gastrointestinal quality of life index

On the gastrointestinal symptom subscale, 105 (63.3 %) patients reported a clinically relevant improvement (Table 3). The emotional subscale showed a clinically relevant improvement in 37 (22.3 %) patients. A clinically relevant improvement of the physical subscale was reported by 54 (32.5 %) patients. Thirty-nine (23.5 %) patients showed a clinically relevant improvement of the social subscale.

**Table 3:** Univariable and multivariable association of pain episode characteristics with patient-reported improvement of health status subscales

	Clinically relevant improvement		Univariable analysis		Multivariable analysis	
	<5 points on GIQLI, *n* = 61	≥5 points on GIQLI, *n* = 105	Odds ratio (95 % CI)	P-value	Odds ratio (95 % CI)	P-value
Gastrointestinal symptoms subscale						
Age (years)	47.1 ± 15.2	50.9 ± 12.7	1.02 (1.00-1.05)	0.087	1.03 (1.00-1.06)	0.048
Sex				0.056		0.230
Female	42 (68.9)	86 (81.9)	2.05 (0.98-4.27)		1.74 (0.71-4.32)	
Male	19 (31.1)	19 (18.1)	1.00 (reference)			
Hospital type				0.143		0.228
Tertiary referral center	17 (27.9)	19 (18.1)	0.57 (0.27-1.21)		0.56 (0.22-1.43)	
Non-academic	44 (72.1)	86 (81.9)	1.00 (reference)			
Baseline GIQLI score	117.9 ± 17.8	95.1 ± 19.9	0.94 (0.92-0.96)	<0.001	0.94 (0.91-0.96)	<0.001
ASA fitness grade				0.148		
II	26 (42.6)	57 (54.3)	1.60 (0.85-3.02)			
I	35 (57.4)	48 (45.7)	1.00 (reference)			
Frequency of pain episodes a week	0.4 (0.2-0.7)	0.4 (0.2-0.8)	1.21 (0.58-2.50)	0.613		
Duration of longest pain episode in hours	4.0 (2.0-8.0)	4.0 (2.0-8.0)	1.01 (0.96-1.05)	0.828		
Maximum intensity of pain episode ranging 0-100	89.0 (77.0-96.5)	94.0 (78.0-100.0)	1.01 (0.99-1.03)	0.329		
Emotional subscale						
Age (years)	49.6 ± 13.8)	49.0 ± 13.7	1.00 (0.97-1.02)	0.790	0.99 (0.95-1.02)	0.389
Sex				0.277		0.568
Female	97 (75.2)	31 (83.8)	1.70 (0.65-4.46)		0.71 (0.22-2.32)	
Male	32 (24.8)	6 (16.2)	1.00 (reference)			
Hospital type				0.991		0.736
Tertiary referral center	28 (21.7)	8 (21.6)	1.00 (0.41-2.42)		1.20 (0.42-3.47)	
Non-academic	101 (78.3)	29 (78.4)	1.00 (reference)			
Baseline GIQLI score	109.7 ± 18.5	81.9 ± 20.2	0.93 (0.91-0.95)	<0.001	0.93 (0.90-0.95)	<0.001
ASA fitness grade				0.852		
II	65 (50.4)	18 (48.6)	0.93 (0.45-1.94)			
I	64 (49.6)	19 (51.4)	1.00 (reference)			
Frequency of pain episodes a week	0.4 (0.2-0.7)	0.4 (0.2-1.0)	1.01 (0.44-2.31)	0.978		
Duration of longest pain episode in hours	4.0 (2.0-8.0)	5.0 (2.0-8.0)	1.02 (0.97-1.08)	0.362		
Maximum intensity of pain episode ranging 0-100	92.0 (77.0-99.0)	95.0 (79.0-99.3)	1.01 (0.99-1.03)	0.524		
Physical subscale						
Age (years)	49.6 ± 14.4	49.3 ± 12.5	0.99 (0.98-1.02)	0.916	0.99 (0.96-1.02)	0.524
Sex				0.353		0.584
Female	84 (75.0)	44 (81.5)	1.47 (0.65-3.29)		0.76 (0.28-2.03)	
Male	28 (25.0)	10 (18.5)	1.00 (reference)			
Hospital type				0.278		0.310
Tertiary referral center	27 (24.1)	9 (16.7)	0.63 (0.27-1.45)		0.61 (0.23-1.59)	
Non-academic	85 (75.9)	45 (83.3)	1.00 (reference)			
Baseline GIQLI score	111.2 ± 19.7)	87.5 ± 17.9	0.94 (0.92-0.96)	<0.001	0.94 (0.92-0.96)	<0.001
ASA fitness grade				1.000		
II	56 (50.0)	27 (50.0)	1.00 (0.52-1.91)			
I	56 (50.0)	27 (50.0)	1.00 (reference)			
Frequency of pain episodes a week	0.38 (0.19-0.67)	0.33 (0.18-1.00)	1.27 (0.62-2.62)	0.514		
Duration of longest pain episode in hours	4.0 (2.0-6.0)	5.0 (2.4-13.5)	1.07 (1.02-1.12)	0.007		
Maximum intensity of pain episode ranging 0-100	91.0 (77.0-100.0)	94.0 (86.0-99.0)	1.01 (0.99-1.03)	0.316		
Social subscale						
Age (years)	47.5 ± 12.6	50.1 ± 14.1	0.99 (0.96-1.01)	0.303	0.97 (0.94-1.01)	0.096
Sex				0.207		0.622
Female	95 (74.8)	33 (84.6)	1.85 (0.71-4.83)		1.40 (0.37-5.28)	
Male	32 (25.2)	6 (15.4)	1.00 (reference)			
Hospital type				0.132		0.069
Tertiary referral center	31 (24.4)	5 (12.8)	0.46 (0.16-1.27)		0.28 (0.07-1.10)	
Non-academic	96 (75.6)	34 (87.2)	1.00 (reference)			
Baseline GIQLI score	109.8 ± 19.7	82.7 ± 15.9	0.93 (0.91-0.96)	<0.001	0.93 (0.91-0.96)	<0.001
ASA fitness grade				0.855		
II	63 (49.6)	20 (51.3)	1.07 (0.52-2.19)			
I	64 (50.4)	19 (48.7)	1.00 (reference)			
Frequency of pain episodes a week	0.33 (0.17-0.63)	0.50 (0.21-1.00)	2.39 (1.11-5.12)	0.025	2.95 (1.08-8.08)	0.035
Duration of longest pain episode (hours)	4.0 (2.0-6.0)	6.0 (3.0-23.0)	1.09 (1.04-1.14)	<0.001	1.10 (1.03-1.17)	0.003
Maximum intensity of pain episode ranging 0-100	91.5 (77.0-98.0)	95.0 (79.3-100.0)	1.01 (0.99-1.03)	0.546		

Data are expressed as mean (standard deviation) or n (%) or median (interquartile range). 95 % CI, 95 % confidence interval; *ASA* American Society of Anesthesiologists, *GIQLI* gastrointestinal quality of life index

Duration was associated with clinically relevant improvement of the physical (OR 1.07, 95 % CI 1.02–1.12; *P* = 0.007) and social subscales (OR 1.09, 95 % CI 1.04–1.14; *P* < 0.001) in univariable analysis (Table 3). Univariable analysis showed frequency to be associated with clinically relevant improvement of the social subscale (OR 2.39, 95 % CI 1.11–5.12; *P* = 0.025). In multivariable analysis, duration (OR 1.10, 95 % CI, 1.03–1.17; *P* = 0.003) and frequency (OR 2.95, 95 % CI 1.08–8.08; *P* = 0.035) of abdominal pain episodes with clinically relevant improvement of the social subscale remained associated.

## Discussion

This study showed a clinically relevant improvement of overall health status in 131 (78.9 %) patients at 12 weeks after cholecystectomy. Episode characteristics of pain were not associated with an overall clinically relevant improvement of health status after surgery, but patients with a higher frequency and a longer duration of abdominal pain episodes were more likely to have a clinically relevant improvement of social functioning after surgery.

The preoperative health status score and improvement of health status were similar to studies using the same patient-reported outcomes.^1^^–^4 We measured patient-reported outcomes at 12 weeks after cholecystectomy as studies suggest that the results at this time point persist at long-term follow-up.^10^,^20^ In studies defining clinically relevant health outcome after surgery exclusively as pain or symptom relief, abdominal pain episode characteristics were associated with a better outcome.^6^–^10^ We showed, however, that characteristics of abdominal pain episodes were not associated with overall health outcome when other factors such as emotional and social functioning are also taken into account. No associations were found between abdominal pain episode characteristics and all the subscales of health status, except for the social subscale. This study showed an association of increased pain episode frequency with the social subscale of health status improvement. In addition, a longer duration of pain episodes has been associated with absence of pain,^10^ whereas an association was found with clinically relevant improvement of social functioning in this study. The higher frequency and longer duration may have been caused by an undetected social disabling mild acute cholecystitis.^13^ This suggestion may certainly fit with the spectrum of cholecystolithiasis.

The main explanation for the discrepant results with literature is the difference in patient-reported outcomes. The most comprehensive patient-reported outcome measure in patients with uncomplicated symptomatic cholecystolithiasis to determine appropriate and efficient utilization of cholecystectomy is still under debate. Postoperative absence of pain, satisfaction, and health status improvement all have been previously used as primary patient-reported outcome measures.^11^ Argument for using patient-reported absence of postoperative pain as primary outcome is that the diagnosis of uncomplicated symptomatic cholecystolithiasis is based on abdominal pain.^21^–^23^ In addition, postoperative pain after cholecystectomy is the main predictor of a patient-reported unsuccessful outcome.^24^ Satisfaction as primary outcome has the advantage of providing information about the relationship between patient expectations and the treatment experience. Satisfaction incorporates the description of healthcare from the patient’s viewpoint, measurement of the process of care, and evaluation of its outcome.^11^ Finally, argument for using health status improvement is that it measures various domains of health and on a continuous scale. This outcome allows us to determine which patient benefits most from therapy.^11^ Health status improvement was therefore chosen as primary patient-reported outcome in this study.

Our study has some limitations. First, we cannot fully rule out recall bias, although we limited this type of bias by excluding patients that could not recall abdominal pain frequency. The generalizability of the results may therefore be limited, although the patient characteristics are no different compared with the characteristics of other studies.^5^–^10^ Second, the inclusion of referred patients and the limited response rate may have caused selection bias, although we did not find any significant differences between the responding and non-responding patients. Third, we performed a post hoc analysis using a database of a previous multicenter cohort study. A formal power analysis was therefore not conducted. Finally, the natural course of symptoms,^25^,^26^ placebo effect of surgery,^27^ or expectancy of patients^28^ may have biased the questionnaire answers. Concerning the wax and waning of abdominal pain episodes,^5^ we corrected for preoperative health status. Unfortunately, randomized trials to limit biased questionnaire answers were prohibited due to ethical reasons.

This study included several strengths as well. First, the database of a prospective observational study was used limiting confounding bias. Second, we used a standardized and validated questionnaire allowing reliable comparisons with other studies using this widely translated and validated questionnaire.^15^,^16^ Third, using a single interviewer in all three centers excluded interobserver bias. Finally, patients were recruited from both tertiary and general hospitals increasing the generalizability.

Since patients that benefit most in terms of health status improvement cannot be predicted using abdominal pain episode characteristics, future studies should assess which uncomplicated symptomatic cholecystolithiasis patients are at increased risk for complicated cholecystolithiasis. Although the risk of complications because of gallstones in uncomplicated symptomatic cholecystolithiasis patients is estimated to be only 1–3 % a year, these complications can be serious and life threatening as previously reported in this journal.^29^,^30^ Preventing uncomplicated symptomatic cholecystolithiasis patients to proceed to complicated symptomatic cholecystolithiasis by early cholecystectomy would increase the cost-effectiveness of this common surgical procedure.

In conclusion, frequency, maximum duration, and intensity of abdominal pain episodes are not associated with a patient-reported clinically relevant improvement of health status at 12 weeks after cholecystectomy. Characteristics of abdominal pain episodes cannot be used to inform patients with symptomatic cholecystolithiasis who are skeptic about the timing of cholecystectomy for optimal benefit. Timing of cholecystectomy for these patients should therefore be based on other characteristics and preferences.
